# Effect of Continuous Ingestion of Bifidobacteria and Dietary Fiber on Improvement in Cognitive Function: A Randomized, Double-Blind, Placebo-Controlled Trial

**DOI:** 10.3390/nu15194175

**Published:** 2023-09-27

**Authors:** Naoki Azuma, Takashi Mawatari, Yasuo Saito, Masashi Tsukamoto, Masatoshi Sampei, Yoshitaka Iwama

**Affiliations:** 1 R&D Laboratory, Ezaki Glico Co., Ltd., 4-6-5, Utajima, Nishiyodogawa-Ku, Osaka 555-8502, Japan; takashi.mawatari@glico.com (T.M.); yasuo.saito@glico.com (Y.S.); masashi.tsukamoto@glico.com (M.T.); masatoshi.sampei@glico.com (M.S.); 2 Nihonbashi Cardiology Clinic, Kyodo Bldg. #201, 13-4 Nihonbashi Kodenmacho, Chuo-Ku, Tokyo 103-0001, Japan; yiwama@well-sleep.jp

**Keywords:** *Bifidobacterium animalis* subsp. *lactis*, probiotics, visceral fat, metabolic syndrome, gut microbacteria, anti-inflammatory, mild cognitive impairment, aging

## Abstract

*Bifidobacterium animalis* subsp. *lactis* GCL2505 has been shown to have some positive effects on health, including improved defecation frequency and reduced visceral fat. These effects are thought to be due to GCL2505′s unique ability to reach the intestine in a viable form and proliferate after a single intake. This leads to an increased number of intestinal bifidobacteria. This randomized, double-blind, placebo-controlled, parallel-group study was conducted to confirm that intake of GCL2505 and inulin (a prebiotic) improve cognitive function (*n* = 80). Participants consumed test drinks containing 1 × 10^10^ colony-forming units of GCL2505 per 100 g and 2.0 g of inulin per 100 g for 12 weeks. The change in cognitive function assessment scores was set as the primary endpoint. There were significant improvements in scores in the neurocognitive index domain, which is an assessment of overall cognitive function, in addition to overall attention, cognitive flexibility, and executive function domains. The intervention significantly increased the number of fecal bifidobacteria and affected the levels of several inflammatory markers. These results suggest that intake of GCL2505 and inulin improves cognitive function by improving the intestinal environment and alleviating inflammation.

## 1. Introduction

It is estimated that more than 55 million people have dementia worldwide, and nearly 10 million new cases of the disease occur each year. Dementia is now the seventh leading cause of death and one of the leading causes of disability and dependency among the elderly. The global economic cost of dementia has reached USD 1.3 trillion, approximately half of which is attributed to care provided by informal caregivers (e.g., family members and close friends), who provide an average of 5 h of care and supervision per day [1]. Furthermore, it is estimated that the number of people with dementia will continue to rise, reaching 78 million by 2030 and 139 million by 2050 [2].

Alzheimer’s disease (AD), a disease of progressive cognitive decline, is the most common form of dementia, with a prevalence of more than 60%, and represents a serious threat to public health [1]. Although the pathogenesis of AD remains to be elucidated, a number of therapeutic approaches have been developed based on the “amyloid cascade hypothesis”, that neurofibrillary tangles are caused by the increase and accumulation of amyloid-β (Aβ) in the brain, followed by abnormal phosphorylation of tau protein [3,4]. AD is a chronic disease of the brain that progresses over decades, with Aβ accumulation in the brain beginning decades before clinical symptoms appear [5]. Once AD develops, it is difficult to improve its symptoms, and currently available AD drugs can only slow the progression of the disease; thus, there is no fundamental cure. For this reason, it is desirable to prevent the onset of AD. Mild cognitive impairment (MCI) is a pre-dementia condition characterized as a cognitive state between normal cognitive aging and dementia and is associated with an increased risk of developing AD [6]. Clinical studies have shown that patients with MCI progress to AD at a rate of 10% to 15% per year [7]. Meanwhile, it has been reported that it is possible to regress from MCI to a cognitively normal state [8,9]. Therefore, implementing appropriate intervention at the MCI stage is critical to reduce the number of patients with dementia. In addition, AD is not only caused by genetic factors and aging but also by environmental factors such as lifestyle habits, including sleep and diet. Accordingly, it is desirable for substances that contribute to the prevention of AD to be included in daily-use foods and used on a routine basis [10].

Various studies have investigated the triggers of AD, clarifying the relationship between obesity and cognitive function as well as the mechanisms of obesity-induced cognitive decline. An observational study reported that cognitive function was lower in participants with more visceral fat [11]. Another study reported that the insulin resistance that developed with obesity promoted the accumulation of Aβ and the formation of neurofibrillary tangles [12]. The association between inflammation and cognitive function has also received attention. Acute and chronic systemic inflammation associated with increased levels of tumor necrosis factor (TNF)-α, a typical inflammation-inducing cytokine, are associated with increased cognitive decline in AD [13]. Thus, management of environmental factors is now considered important for the prevention of AD, and lifestyle interventions aimed at preventing the onset and progression of AD as well as preventive measures in daily life with functional foods are necessary as one approach to solving the problem [14]. In addition, food ingredients such as chlorogenic acid [15], propolis extract [16], and astaxanthin [17] have been reported to improve cognitive function, while probiotics are expected to play a major role in future dementia countermeasures, based on the results of meta-analyses showing that probiotics improve cognitive function in patients with MCI [18]. The cognitive improvement effect of the probiotic bacterial strain *Lactobacillus rhamnosus* GG might be attributable to the improvement of signaling markers [19]. Meanwhile, intake of *Bifidobacterium breve* A1 might contribute to the modulation of brain immune response through the production of short-chain fatty acids (SCFAs), thereby contributing to the improvement of cognitive function [20,21]. It has also been reported that intake of *Lactiplantibacillus plantarum* OLL2712 protects against memory decline in the elderly due to its high IL-10 induction activity in immune cells [22].

*Bifidobacterium animalis* subsp. *lactis* GCL2505, a probiotic strain originally isolated from the feces of healthy adults [23,24,25], has been shown to reduce visceral fat [26]. Horiuchi et al. reported that GCL2505 affects host metabolic homeostasis (e.g., enhanced glucose tolerance, suppressed body fat accumulation) in a GPR43-dependent manner, due to enhanced SCFA production in the gut [27]. In clinical trials, daily consumption of fermented milk containing GCL2505 was shown to reduce abdominal visceral fat mass [28]. Furthermore, clinical studies have shown that GCL2505, when taken in combination with inulin [29], a typical prebiotic material, increases the total number of bifidobacteria in the gut more than GCL2505 alone [30].

In our study based on previous results, it was newly found that the combined intake of GCL2505 and inulin may reduce the risk of cognitive decline via visceral fat reduction. We also speculate that the anti-inflammatory effect of acetic acid produced by GCL2505 in the gut might improve cognitive function. SCFAs are known to exhibit anti-inflammatory effects by modulating immune cell chemotaxis as well as the release of reactive oxygen species (ROS) and cytokines [31]. However, it has not yet been demonstrated that the visceral-fat-reducing and anti-inflammatory effects of probiotics contribute directly to improving cognitive function. Therefore, in the present study, we conducted a randomized, double-blind, placebo-controlled, parallel-group study to test the hypothesis that the combined intake of GCL2505 and inulin improve cognitive function.

## 2. Materials and Methods

### 2.1. Participants

Participants were Japanese men and women between the ages of 50 and 80 years at the time of consent, who satisfied the inclusion criteria, did not satisfy any of the exclusion criteria, and were deemed eligible to participate by the study investigator. In this study, participants had to be selected from healthy people, not sick people, because the effects of food consumption—bifidobacteria and inulin—had to be confirmed. Young adults with documented cognitive decline were excluded from the study because they were more likely to have AD or other illnesses. Participants had to be drawn from healthy individuals with mild cognitive decline due to aging. The inclusion criteria were as follows: (1) score of 24 or higher on the Mini Mental State Examination—Japanese (MMSE-J), 17 or higher on the Japanese version of the Montreal Cognitive Assessment (MoCa-J), and 5 or less on the Geriatric Depression Scale—short version—Japanese (GDS-S-J); (2) subjective symptoms of memory loss or reported by close relatives or acquaintances to have other symptoms of memory loss; and (3) fully informed of the purpose and content of the study, deemed to have the capacity to consent, and volunteered of their own accord to participate in the study based on a thorough understanding of the purpose and content of the study, and provided written informed consent to participate in the study. Exclusion criteria were as follows: (1) current or past history of mental disorders (including depressive symptoms), cerebrovascular diseases, and sleep disorders; (2) serious liver, kidney, heart, respiratory, endocrine, or metabolic diseases; (3) smoker; (4) regular alcohol user (consuming ≥ 60 g pure alcohol equivalent daily) with an extremely irregular diet; (5) unable to follow the restrictions on foods or supplements that affect the intestinal environment during the study period; (6) use of antibiotics within 1 month prior to the start of the study; (7) have undergone digestive surgery (excluding appendicitis); (8) have experienced allergies to any of the study food ingredients; (9) currently taking medications that may affect cognitive function (e.g., antipsychotic, anxiolytic, antidepressant, antiparkinsonian, antisomatics, antiepileptic, and anticoagulant medications); (10) visual or hearing impairment that may interfere with cognitive function tests; (11) routinely consume foods or supplements that may affect cognitive function; (12) current or former drug or alcohol dependence; (13) participation in research involving the ingestion of other foods or the use of pharmaceuticals, the application of cosmetics or pharmaceuticals, or participation in other research while participating in this study; and (14) deemed ineligible by the principal investigator.

### 2.2. Test Foods

The test products were a dairy drink (active drink) containing inulin (Orafti GR; BENEO GmbH, Mannheim, Germany) and GCL2505 or placebo. The active drink contained 1 × 10^10^ colony-forming units of GCL2505 and 2.0 g of inulin per 100 g. The placebo was prepared using the same ingredients as the active drink, with the addition of food-grade acetic acid and lactic acid to adjust the flavor and pH; the basic ingredients were skim milk powder, fructose, dextrose, sucrose, yeast extract, acidifier, stabilizer, and flavoring. The nutritional details of the test products are shown in Table 1.

### 2.3. Experimental Design

This was a randomized, placebo-controlled, double-blind, parallel-group study. Participants were stratified by age at screening and sex, and Cognitrax (short), MMSE-J, and MoCa-J scores served as stratification factors for randomizing in block sizes of four by computer-generated randomization to two groups (1:1). The controller (allocation manager) assigned the two groups to the test drink intake group and the control food intake group. For sample size, the final target number of subjects was set at 80, referring to previous reports on probiotic-induced cognitive function [21,22]. Participants in the active and placebo groups consumed 100 g of dairy beverage once daily for 12 weeks. Both the participants and observers were blinded to the group allocation for the duration of the study. Double blinding was accomplished by labeling the test drink with only an identification number. The change in Cognitrax (long) scores between weeks 0 and 12 was set as the primary endpoint. The secondary endpoints were Cognitrax (long) scores between weeks 0 and 8, fecal bifidobacteria, SF-36v2^®^ scores, blood inflammation markers (Olink Target 96 Inflammation), blood high-sensitivity C-reactive protein (*hs*-CRP), blood interleukin (IL)-1β, and serum brain-derived neurotrophic factor (BDNF). The study was conducted at Nihonbashi Cardiology Clinic (Tokyo, Japan) from September to December 2022 by K.S.O. Corporation (Tokyo, Japan), a contract research organization, and was registered with the University Hospital Medical Information Network Clinical Trials Registry (UMIN-CTR) “http://www.umin.ac.jp/ctr/index.htm (accessed on 15 July 2022)” as UMIN000048386. This article conforms to the Consolidated Standards of Reporting Trials (CONSORT) 2010 guidelines (Supplementary Materials, Table S1).

### 2.4. Cognitrax Test

Participants’ cognitive function was measured using Cognitrax, a computer-based battery of cognitive function tests that was developed as a Japanese version of CNS Vital Signs [32]. Based on a previous study [33], the cognitive function tests were administered in the following order: verbal memory test, visual memory test, finger tapping test, symbol digit coding test, Stroop test, shift attention test, continuous performance test, perception of emotion test, nonverbal reasoning test, and four-part continuous performance test.

### 2.5. Quality of Life Test

The SF-36v2^®^, a widely used quality of life rating scale, consists of the following eight scales: “physical functioning”, “role physical”, “bodily pain”, “general health perceptions”, “vitality”, “social functioning”, “role emotional”, and “mental health”. The score for each scale was estimated based on national norms (norm-based scoring) and calculated as a standard score (mean, 50) [34].

### 2.6. Mental Health Status

The Japanese version of the World Health Organization Five Well-Being Index (WHO-5-J) survey was conducted at weeks 0, 8, and 12 [35] to confirm that participants had no abnormal mental health status during the study period.

### 2.7. Biochemical Parameters

Blood pressure, pulse rate, and body weight were measured at weeks 0, 8, and 12. The concentrations of biochemical parameters were also measured at weeks 0 and 12. Blood samples were drawn from each participant after 4 h of no food or drink except water (fasting) prior to testing. Biochemical parameters included hematological tests (white blood cell count, red blood cell count, hemoglobin, hematocrit, mean corpuscular volume, mean corpuscular hemoglobin, mean corpuscular hemoglobin concentration, platelet count, leukogram), biochemical tests (total protein, albumin, total bilirubin, aspartate aminotransferase, alanine aminotransferase, lactate dehydrogenase (IFCC), alkaline phosphatase (IFCC), gamma-glutamyltransferase, urea nitrogen, creatinine, uric acid, sodium, chlorine, potassium, calcium, total cholesterol, low-density lipoprotein cholesterol, high-density lipoprotein cholesterol, triglycerides, phospholipids, glucose, HbA1c (NGSP), insulin), and urinalysis (protein, sugar, bilirubin, urinary ketone bodies, occult blood reaction, urobilinogen, pH, and specific gravity). In addition, serum BDNF was quantified. All of these tests were performed at LSI Medience Corporation (Tokyo, Japan).

### 2.8. Inflammatory Protein Profile

Blood inflammation markers in frozen serum were determined using Olink^®^ Target 96 Inflammation Panels (Olink Proteomics AB, Uppsala, Sweden) with proximity expansion technology, a high-throughput multiplex proteomic immunoassay [36]. The panel contains 92 immune-related proteins, mostly cytokines and chemokines. The assay involves epitope-specific binding and hybridization of a set of paired oligonucleotide antibody probes, followed by amplification using quantitative PCR, normalized on a log base 2 to Olink’s own relative abundance units (normalized protein expression values). Quality control was performed on samples (using spiked internal controls) and external controls for each sample plate. This inspection was performed by Pharma Foods Corporation (Tokyo, Japan). In addition, blood *hs*-CRP (LSI Medience Corporation) and blood IL-1β (Filgen, Inc., Aichi, Japan) were quantified.

### 2.9. Fecal Samples

Fecal samples were submitted at weeks 0 and 12. Fecal samples were collected at home between 7 and 2 days before the specified visit. The submitted samples were promptly transported to the laboratory by refrigerated transport.

### 2.10. Fecal DNA Extraction

Bacterial DNA was extracted from fecal samples using the ISOSPIN Fecal DNA Kit (Nippon Gene Co., Ltd., Tokyo, Japan), following the procedure of Tourlousse et al. [37]. Specifically, a sample (here, 0.2 g fecal sample), 700 μL of FE1 buffer, and 10 μL of RNase were added to a tube with attached beads. A bead-beating homogenizer (FastPrep-24; MP Biomedicals, Irvine, CA) was used at a rate of 6 m/s for 1 min to crush the cells. The process was repeated three times, during which the sample was kept at room temperature for 5 min. Then, 90 µL of FE2 buffer was added and the samples were centrifuged at 12,000× *g* for 15 min. The supernatant (up to 500 µL) was collected and mixed with FB buffer and isopropanol, each at 0.4× the volume of the supernatant obtained. Finally, the sample was loaded onto a spin column and washed according to the manufacturer’s instructions. Purified DNA was eluted with 50 µL of Tris-EDTA buffer (pH 8.0).

### 2.11. Fecal Bifidobacteria

Following Tanaka et al. [38], real-time polymerase chain reaction (PCR) was performed using genus-specific primers capable of detecting *Bifidobacterium* spp., including GCL2505. The primer sequences were as follows: *Bifidobacterium* spp. sense primer, 5′-GATTCTGGCTCAGGATGAACGC-3′; *Bifidobacterium* spp. antisense primer, 5′-CTGATAGGACGCGACCCCAT-3′. Each PCR reaction mixture consisted of 20 pmol of each primer in a total volume of 10 µL; 5 µL of SYBR^®^ premix Ex taq (Takara Bio, Shiga, Japan); and 1 µL of DNA solution [38]. This inspection was performed by the Kyoto Institute of Nutrition and Pathology (Kyoto, Japan).

### 2.12. Statistical Analysis

All measurements were expressed as mean and standard deviation (SD). All statistical analyses were performed using IBM^®^ SPSS^®^ Statistics 27 (IBM Corp., Armonk, NY, USA). A *p*-value < 0.05 was used as the threshold for determining significance. As basic statistics, means and SDs are expressed to the nearest significant digit, percentages are expressed to one decimal place, and finally digits were adjusted by rounding. Missing data were treated as missing values and no surrogate values were used; Cognitrax (Long) statistical analyses were performed with paired *t*-tests in order to compare the test results at the start date of the intake (week 0) with those at 8 and 12 weeks after intake. For the other items, Dunnett’s test (two-tailed) was used to compare the test results at the start of intake (week 0) with those at 8 and 12 weeks after the start of intake, and the Wilcoxon signed rank test was used for qualitative items. Comparisons between the active and placebo groups at each examination time were statistically analyzed with an unpaired *t*-test (two-tailed), and a Wilcoxon’s rank-sum test was used to compare the qualitative endpoints.

## 3. Results

### 3.1. Analysis of the Participant Population

**Figure 1:**
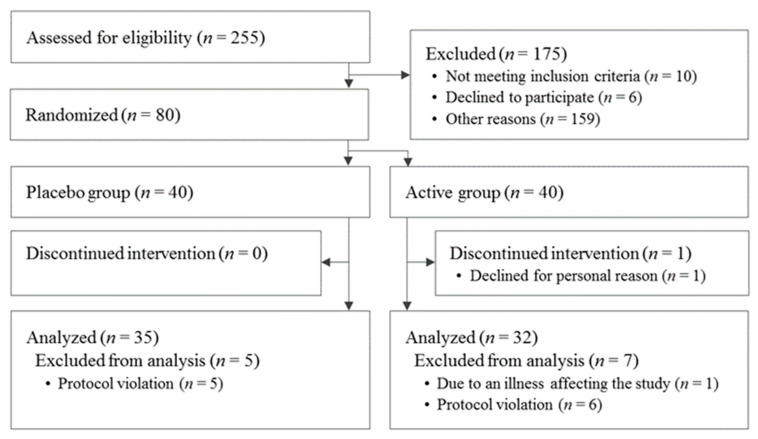
Flowchart of participant selection.

The participant selection process is shown in Figure 1. A total of 255 participants were screened for this study. After screening, 80 participants were eligible: 40 were assigned to the active group and 40 were assigned to the placebo. A significant difference in alkaline phosphatase levels between the active and placebo groups was observed at the beginning of the study but was deemed acceptable because it was within the reference range. For the other items, there were no differences in the baseline characteristics of the participants’ data (Table 2). By the end of the study, one participant from the active group withdrew for personal reasons. After the completion of the entire study, one participant from the placebo group was dropped due to an extremely irregular lifestyle. One participant from the active group was dropped due to a confirmed illness unrelated to the study that may have affected the results. In addition, nine participants were also excluded because they were found to have consumed drugs or foods during the study period that might have affected the results (*n* = 5 from the active group and *n* = 4 from the placebo group). Another participant in the active group was dropped due to partial missing primary-endpoint data. Thus, a total of 67 patients (32 in the active group and 35 in the placebo group) were included in the analysis. Moreover, four participants with reduced WHO-5-J scores were also excluded for Cognitrax analysis (*n* = 1 from the active group and *n* = 3 from the placebo group) because they had a score of ≤50, which is used as the cut-off for assigning a ‘screening diagnosis’ of depression in the global version of WHO-5 [39]; reduced WHO-5-J scores might indicate the possibility of earlier depression caused by isolation due to the COVID-19 pandemic, and thus Cognitrax tests may not have been performed properly in these participants. There were no reported harms or unintended effects in each group.

### 3.2. Cognitrax Test

The change in neurocognitive index domain score from week 0 to week 12 in the active group (5.5 ± 7.1) was greater than that in the placebo group (2.3 ± 4.0), and there was a statistically significant difference between them (*p* = 0.027 by the unpaired *t*-test). Furthermore, the changes in complex attention domain score (8.3 ± 11.8 vs. 3.2 ± 6.9, *p* = 0.041 by the unpaired *t*-test), cognitive flexibility domain score (9.8 ± 11.5 vs. 4.8 ± 6.7, *p* = 0.038 by the unpaired *t*-test), and executive function domain score (9.5 ± 11.8 vs. 4.5 ± 6.9, *p* = 0.044 by the unpaired *t*-test) in the active group from week 0 to 12 were significantly higher than those in the placebo group (Table 3). The results of the Cognitrax task to calculate scores for each domain are presented in Supplementary Materials, Table S2.

### 3.3. Quality of Life Test

Changes in quality of life during the study period were assessed by the SF-36v2^®^. No statistically significant differences were found between the active and placebo groups in terms of change in score (Table 4).

### 3.4. Fecal Bifidobacteria

**Figure 2:**
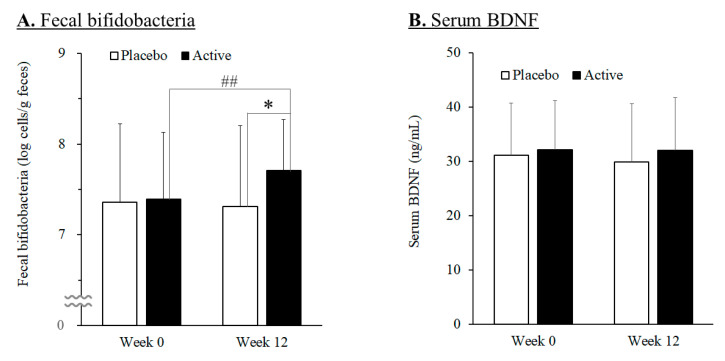
Changes in fecal bifidobacteria (**A**) and in serum BDNF (**B**) in the placebo (*n* = 35) and active (*n* = 32) groups during the study period. Values are means, with error bars as standard deviation. Double number signs (##) indicate *p*-value < 0.01 as a result of intra-group difference (week 0 vs. week 12; paired *t*-test). Asterisk (*) indicates *p*-value < 0.05 as a result of inter-group difference (the placebo group vs. the active group; unpaired *t*-test).

Quantification of the bifidobacteria in the feces (Figure 2A) revealed that the total number of bifidobacteria in the active group at week 12 increased significantly compared to week 0, while that of the placebo group did not change much. And the total number of bifidobacteria was significantly increased in the active group (7.71 ± 0.56 log cells/g feces) compared with the placebo group (7.31 ± 0.90 log cells/g feces) at week 12 (*p* = 0.031 using the unpaired *t*-test).

### 3.5. Serum BDNF

Serum BDNF levels were quantified. The results showed no statistically significant difference between the active and placebo groups during the study period (Figure 2B).

### 3.6. Blood Inflammation Markers

The expression levels of 92 inflammatory markers were examined at weeks 0 and 12 using Olink^®^ Target 96 Inflammation Panels; 75 inflammatory markers that were determined to be quantifiable were analyzed (Supplementary Materials, Table S3). The results showed that there were statistically significant differences between the change in scores from week 0 to week 12 for leukemia inhibitory factor receptor, sulfotransferase 1A1, C-C motif hemokine (CCL)23, and TNF (ligand) superfamily member 12 (TWEAK) in the active and the placebo groups (using the unpaired *t*-test) (Table 5). In addition, there were no statistically significant differences between the change in scores from week 0 to week 12 of adenosine deaminase, osteoprotegerin, eotaxin, glial cell line-derived neurotrophic factor, fractalkine, interleukin-8 (IL-8), CCL28, IL-18, IL-10, CCL19, and T-cell surface glycoprotein CD5 in the active and placebo groups, but the trends were considered significant because the *p*-value was <0.1 (Table 5). Blood *hs*-CRP and blood IL-1β were also measured at weeks 0 and 12, but neither showed a statistically significant difference between the active and placebo groups (Supplementary Materials, Table S4).

## 4. Discussion

We investigated the effects of consuming a dairy beverage containing *Bifidobacterium animalis* subsp. *lactis* GCL2505 and inulin on cognitive function in healthy adults. The results showed that the test drink had a positive effect on cognitive function.

Cognitive function was assessed using Cognitrax, a computerized battery of neurocognitive tests developed for routine clinical screening applications. Cognitrax has been reported to have very similar characteristics to traditional psychological tests. It can measure a wide range of cognitive functions and is suitable for accurate assessment of scored cognitive functions because of its high sensitivity to discriminating between individuals with MCI and healthy individuals [32]. A similar test, CNS Vital Signs, is reported to adequately discriminate between healthy individuals, patients with MCI, and patients with dementia [40]. Cognitrax has also been used to assess cognitive function in clinical trials [15,16,17]. Therefore, Cognitrax was considered a suitable tool for testing cognitive function in this study.

The change in neurocognitive index domain scores from week 0 to week 12 was significantly higher in the active group than in the placebo group. The Neurocognitive Index domain score is calculated by averaging the scores from the total memory, cognitive functioning speed, reaction time, total attention, and cognitive flexibility domains, and is, therefore, used to assess a person’s overall neurocognitive status. Furthermore, intake of GCL2505 and inulin was associated with statistically significant improvements in scores in the complex attention, cognitive flexibility, and executive function domains. Total attention refers to the ability to process things accurately while maintaining attention. Cognitive flexibility refers to the ability to understand and process changes in instructions. Executive function refers to the ability to make decisions based on an understanding of background rules and concepts. Thus, it is considered that intake of GCL2505 and inulin may contribute to improved activities of daily living by improving these functions.

It is hypothesized that intake of GCL2505 and inulin improves cognitive function through a mechanism involving the following three steps. Step 1: Intake of GCL2505 and inulin improves the intestinal environment. In this study, the number of fecal bifidobacteria was significantly increased in the active group compared with the placebo group. It was reported that intake of GCL2505 increases the number of bifidobacteria in the feces and the concentration of acetic acid in the feces and blood [23,25,26,27]. Clinical studies have shown the effects of inulin [41] as well as the enhanced effect of GCL2505 in combination with inulin in terms of increased total bifidobacteria count in the intestine [30]. Thus, participants in the active group who consumed a test drink containing GCL2505 and inulin in the present study had an improved intestinal environment via increasing the number of bifidobacteria in the gut. This change may possibly have increased the level of acetic acid, an SCFA, in the gut of the active group. Step 2: Improvement in the intestinal environment leads to alleviation of inflammation. Increased SCFA levels in the gut alleviate inflammation in the body. It was reported that administration of dietary fiber or acetic acid to mice reduced blood levels of IL-1, a known inflammatory cytokine [42]. SCFAs are also known to have positive effects on inflammation by reducing visceral fat and improving glucose metabolism. Experiments with mice showed that GCL2505 played a role in reducing visceral fat area by increasing the number of bifidobacteria in the intestine, as evidenced by the increased numbers in the feces as well as the higher acetic acid levels in feces and blood [26]. Daily consumption of yogurt containing GCL2505 has been shown in clinical studies to reduce visceral fat mass in the abdomen of humans [28]. Inulin consumption has also been proven to reduce visceral fat area [43], and Lauridsen et al. found that obese individuals have decreased expression of IL-10, which contributes to reducing inflammation, and increased expression of nitric oxide synthase 2, which triggers inflammation, in the brain [44]. A correlation between high-fat diet intake and hypothalamic inflammatory status has been reported [45,46], and Mao et al. reported that administration of a high-fat diet in combination with (-)-epigallocatechin gallate in mice suppressed the increased expression of IL-6, TNF-α, and IL-1β in the hypothalamus by inhibiting body weight gain [47]. Step 3: Alleviating inflammation improves cognitive function. It has recently been shown that inflammatory conditions and cognitive function are closely linked. Studies on patients with AD have reported a correlation between attenuated cognitive function and acute and chronic systemic inflammation associated with increased TNF-α levels [13], and that reduced levels of NLRP3 (nucleotide-binding oligomerization domain-like receptor family, pyrin domain-containing 3) inflammasome-derived inflammatory cytokines alleviate the progression of AD pathology [48]. TNF-α is a typical pro-inflammatory cytokine [49], and Habbas et al. suggested a link between increased TNF-α levels in the brain and cognitive impairment [50]. Increasing the number of SCFA-producing bacteria by fecal transplantation in rats resulted in increased SCFA levels in the gut and a reduction in cognitive decline [51]. The hypothesis thus far indicates that the effects of GCL2505 and inulin on cognitive function may be realized by increasing intestinal SCFA levels and alleviating inflammatory conditions.

In this study, the changes in expression of 75 inflammation markers from week 0 to 12 were analyzed by principal component analysis and tested by permutational multivariate analysis of variance, but there were no statistically significant differences between the active and placebo groups (*p* = 0.109) (Supplementary Materials, Figure S1). However, some reductions in pro-inflammatory cytokine levels were observed. For example, TWEAK is expressed in animals with chronic intestinal inflammation [52] and induces secretion of the pro-inflammatory cytokine IL-8 [53]. CCL23 is also secreted by neutrophils stimulated by lipopolysaccharides and TNF-α [54]. These results suggest that the chronic inflammatory state of the intestinal tract may be somehow affected by GCL2505 and inulin and that the inhibitory effect of *Bifidobacterium breve* A1 on brain atrophy may be related to the suppression of inflammation [55]. The effect of *Lactiplantibacillus plantarum* OLL2712, which has been shown to strongly induce IL-10 production and have an effect on chronic inflammation, was confirmed to improve cognitive function, suggesting that the suppressive effect of OLL2712 on gut and nerve inflammation may be the reason for the improvement [22]. Intake of *Bifidobacterium longum* BB68S led to a decrease in the numbers of the inflammation-inducing bacteria *Solobacterium* and *Oribacterium*, indicating that improved cognitive function might be due to reduced inflammation [56]. Akbari et al. reported that administration of multiple probiotics had a favorable effect on *hs*-CRP as well as MMSE scores, malondialdehyde, insulin metabolic markers, and triglyceride levels in patients with AD [57]. An intervention involving the intake of *Bifidobacterium bifidum* BGN4 and *Bifidobacterium longum* BORI decreased intestinal levels of inflammatory bacteria and increased BDNF levels, suggesting that these bifidobacteria might have an anti-inflammatory effect [58]. The effects of probiotics on cognitive function have also been verified by multiple clinical trials. Many of these effects are presumed to be due to the alleviation of inflammation, but in many cases, the results from indirectly assessing inflammatory conditions have been used. In contrast, in the present study, multiple inflammation markers were measured for the first time in a study in which probiotics were ingested, thereby directly demonstrating the relationship between inflammation and the effect of probiotics on cognitive function. Although further research is needed to understand the inflammatory state, it is believed that the effects of GCL2505 and inulin on cognitive function are strongly related to the inflammatory state in the gut.

The results of the SF-36v2^®^ health-related quality of life assessment in the present study did not reveal any differences between the active and placebo groups. All scores at week 0 were above 50 except for the bodily pain scale. It is possible that the participants in this study did not originally have low SF-36v2^®^ scores, so the improvement effect was not apparent. The selection criteria “subjective or other symptoms of forgetfulness” established in this study suggested that the person’s quality of life was not significantly impaired. Also, we did not confirm the effect of GCL2505 and inulin on BDNF, which is a neurotrophic factor that is essential for synaptogenesis, plasticity, and neuroimmune responses and plays an important role in learning, memory formation, and affective disorders [59,60]. Although BDNF levels have been reported to be associated with gut microbiota [61,62], meta-analyses have shown no correlation between probiotic intake and BDNF levels. [63]. Given the inconsistent relationship between BDNF and the gut microbiota observed here, further research is warranted.

Just to be sure, no serious health problems have been reported as a result of the consumption of foods containing GCL2505. In addition, foods using *Bifidobacterium animalis* subsp. *lactis* have been sold all over the world, and no health hazard has been reported to be caused by these bifidobacteria. So the safety of GCL2505 is assured, but in some cases, intake of GCL2505 may cause slightly increased farting or softer stools in some people.

## 5. Conclusions

This is the first randomized controlled trial to demonstrate the efficacy of GCL2505 and inulin in improving memory function in the elderly. Elderly patients with early memory loss who consumed GCL2505 and inulin for 12 weeks showed significant improvements in scores in the neurocognitive index domain, which is an assessment of overall cognitive function, in addition to the complex attention, cognitive flexibility, and executive function domains, and the number of bifidobacteria in feces increased significantly. Because there is currently no effective pharmacological therapy to prevent the onset and progression of cognitive decline in the pre-dementia stage, the findings of this study suggest that continuous intake of GCL2505 and inulin may be an effective approach to protect memory function in the elderly.

## Figures and Tables

**Table 1:** Nutritional details of the test drinks.

	Placebo	Active
Energy, kcal/100 g	48.0	52.0
Moisture, g/100 g	86.9	84.9
Protein, g/100 g	2.8	2.8
Fat, g/100 g	0.1	0.1
Carbohydrate, g/100 g	9.1	11.2
Ash, g/100 g	1.1	1.1

The active drink contained 2.0 g of inulin and 1.0 × 10^10^ colony-forming units of GCL2505.

**Table 2:** Baseline characteristics of the participants (placebo group: *n* = 40; active group: *n* = 40).

	Placebo Group	Active Group	*p*-Value
Age, years	62.7 (6.9)	64.6 (7.1)	0.229
MMSE-J	28.0 (1.6)	28.0 (1.3)	0.878
Corrected MOCA-J	22.9 (1.8)	22.9 (2.1)	0.955
GDS-S-J	2.1 (1.6)	1.8 (1.7)	0.372
Height, cm	161.5 (9.0)	161.8 (8.0)	0.907
Body weight, kg	59.0 (11.3)	59.5 (12.1)	0.842
Body mass index, kg/m^2^	22.4 (2.8)	22.6 (3.1)	0.861
Systolic blood pressure, mmHg	129.5 (17.0)	128.3 (16.3)	0.733
Diastolic blood pressure, mmHg	77.8 (12.8)	75.7 (10.7)	0.439
Heartbeat, bpm	70.6 (10.7)	71.1 (12.0)	0.830
White blood cell count, /µL	5817.5 (1731.7)	5685.0 (1543.8)	0.719
Red blood cell count, ×10⁴/µL	446 (40.9)	445.5 (41.1)	0.957
Hemoglobin, g/dL	13.7 (1.3)	13.7 (1.1)	0.978
Hematocrit, %	43.3 (3.7)	43.4 (3.3)	0.914
Platelet count, ×10⁴/μL	24.6 (5.1)	23.5 (5.1)	0.354
Mean corpuscular volume, fL	97.3 (4.3)	97.6 (3.8)	0.764
Mean corpuscular hemoglobin, pg	30.9 (1.8)	30.9 (1.3)	0.966
Mean corpuscular hemoglobin concentration, %	31.7 (1.1)	31.7 (0.9)	0.711
Neutrophil ratio, %	54.9 (7.7)	55.4 (8.8)	0.777
Lymphocyte ratio, %	35.1 (6.7)	34.2 (7.7)	0.573
Monocyte ratio, %	6.3 (1.8)	6.0 (1.5)	0.501
Eosinophil ratio, %	2.9 (2.0)	3.6 (2.5)	0.173
Basophil ratio, %	0.8 (0.3)	0.8 (0.3)	0.461
Total serum protein, g/dL	7.1 (0.4)	7.1 (0.4)	0.358
Albumin, g/dL	4.4 (0.2)	4.4 (0.2)	0.718
Aspartate aminotransferase, U/L	22.0 (6.2)	22.2 (9.6)	0.923
Alanine aminotransferase, U/L	18.1 (9.0)	17.5 (9.1)	0.777
Lactate dehydrogenase, U/L	185.1 (23.8)	187.5 (23.5)	0.655
Total bilirubin, mg/dL	1.0 (0.4)	0.9 (0.3)	0.175
Alkaline phosphatase, U/L	59.8 (14.6)	66.1 (13.0)	0.045
γ-Glutamyl transpeptidase, U/L	26.3 (13.7)	30.4 (32.7)	0.467
Blood urea nitrogen, mg/dL	14.8 (2.5)	14.4 (3.4)	0.594
Creatinine, mg/dL	0.8 (0.2)	0.8 (0.1)	0.788
Uric acid, mg/dL	5.6 (1.6)	5.1 (1.3)	0.181
Sodium (Na), mEq/L	141.5 (1.7)	141.2 (1.9)	0.392
Chlorine (Cl), mEq/L	104.3 (2.0)	104.1 (2.1)	0.664
Potassium (K), mEq/L	4.3 (0.4)	4.3 (0.3)	0.667
Calcium (Ca), mg/dL	9.3 (0.3)	9.3 (0.3)	0.966
Total cholesterol, mg/dL	216.3 (35.0)	212.7 (36.0)	0.649
LDL cholesterol, mg/dL	125.0 (30.8)	118.9 (25.6)	0.338
HDL cholesterol, mg/dL	71.3 (17.0)	73.1 (24.1)	0.705
Triglycerides, mg/dL	98.8 (49.1)	103.0 (52.6)	0.711
Phospholipid, mg/dL	233.9 (31.5)	233.9 (37.9)	0.995
Glucose, mg/dL	88.6 (8.4)	87.5 (9.8)	0.601
HbA1c (NGSP), %	5.5 (0.3)	5.5 (0.3)	0.778
Urine pH	6.2 (0.7)	6.2 (0.8)	0.759

All data are presented as mean (standard deviation). Comparisons between the placebo and active groups were tested by analysis of variance.

**Table 3:** Post-intervention changes in each cognitive function parameter (placebo group: *n* = 32; active group: *n* = 31).

		Week 0	Week 8		Week 12	
		Mean (SD)	Mean (SD)	*p*-Value	Mean (SD)	*p*-Value
Neurocognitive index	Placebo	103.5 (5.7)	104.8 (4.9)	0.090	105.7 (5.9)	0.003
	Active	101.6 (6.8)	104.5 (8.2)	0.045	107.2 (5.1)	<0.001
∆ Neurocognitive index	Placebo		1.4 (4.4)	0.345	2.3 (4.0)	0.027
	Active		2.9 (7.6)		5.5 (7.1)	
Composite memory	Placebo	105.3 (13.6)	102.7 (15.9)	0.264	102.6 (12.3)	0.231
	Active	104.4 (14.8)	107.6 (13.6)	0.147	107.2 (13.3)	0.297
∆ Composite memory	Placebo		−2.7 (13.2)	0.070	−2.8 (12.7)	0.114
	Active		3.2 (12.1)		2.8 (14.7)	
Verbal memory	Placebo	105.3 (13.6)	106.1 (15.0)	0.746	105.1 (13.2)	0.926
	Active	104.6 (14.3)	107.9 (13.6)	0.129	108.5 (14.6)	0.142
∆ Verbal memory	Placebo		0.8 (14.1)	0.452	−0.2 (13.3)	0.241
	Active		3.3 (11.7)		3.9 (14.5)	
Visual memory	Placebo	103.7 (14.8)	98.3 (15.7)	0.035	99.2 (14.8)	0.124
	Active	103.3 (12.8)	105.1 (11.8)	0.495	103.7 (11.0)	0.861
∆ Visual memory	Placebo		−5.4 (14.0)	0.047	−4.5 (16.1)	0.202
	Active		1.8 (14.3)		3.9 (14.5)	
Psychomotor speed	Placebo	106.3 (8.8)	108.5 (9.3)	0.045	108.3 (10.1)	0.073
	Active	106.2 (8.3)	108.9 (8.4)	0.016	108.6 (7.6)	0.054
∆ Psychomotor speed	Placebo		2.1 (5.7)	0.664	1.9 (5.8)	0.720
	Active		2.8 (6.1)		2.5 (6.9)	
Reaction time	Placebo	94.7 (11.3)	94.8 (11.8)	0.906	98.8 (11.1)	<0.001
	Active	96.6 (9.1)	98.8 (10.6)	0.112	100.8 (9.5)	0.003
∆ Reaction time	Placebo		0.1 (6.0)	0.230	4.2 (4.8)	0.981
	Active		2.2 (7.3)		4.2 (7.1)	
Complex attention	Placebo	108.3 (8.9)	111.9 (6.2)	0.028	111.5 (6.4)	0.015
	Active	104.0 (11.2)	105.2 (23.4)	0.762	112.3 (4.7)	<0.001
∆ Complex attention	Placebo		3.6 (8.8)	0.580	3.2 (6.9)	0.041
	Active		1.2 (22.4)		8.3 (11.8)	
Cognitive flexibility	Placebo	102.8 (9.3)	106.1 (7.3)	0.004	107.5 (8.4)	<0.001
	Active	96.7 (10.9)	102.2 (9.8)	0.014	106.5 (6.4)	<0.001
∆ Cognitive flexibility	Placebo		3.3 (6.0)	0.359	4.8 (6.7)	0.038
	Active		5.5 (11.6)		9.8 (11.5)	
Processing speed	Placebo	114.4 (9.3)	115.9 (9.9)	0.312	116.8 (10.2)	0.100
	Active	114 (9.3)	117.2 (11.1)	0.058	118.4 (9.3)	0.004
∆ Processing speed	Placebo		1.4 (7.9)	0.403	2.3 (7.7)	0.291
	Active		3.3 (9.2)		4.4 (7.8)	
Executive function	Placebo	102.8 (9.3)	105.3 (7.5)	0.016	107.2 (9)	0.001
	Active	96.8 (11.1)	102.0 (9.9)	0.022	106.3 (6.4)	<0.001
∆ Executive function	Placebo		2.5 (5.5)	0.265	4.5 (6.9)	0.044
	Active		5.2 (11.9)		9.5 (11.8)	
Social acuity	Placebo	86.2 (14.7)	92.0 (14.5)	0.052	94.0 (18.0)	0.013
	Active	90.0 (17.6)	94.5 (17.2)	0.213	98.1 (14.9)	0.018
∆ Social acuity	Placebo		5.8 (16.3)	0.790	7.8 (16.8)	0.937
	Active		4.6 (20.1)		8.2 (18.1)	
Reasoning	Placebo	94.7 (16.5)	91.3 (18.3)	0.264	95.0 (17.5)	0.929
	Active	92.8 (15.9)	96.6 (14.5)	0.166	93.9 (14.2)	0.706
∆ Reasoning	Placebo		−3.4 (16.9)	0.078	0.3 (15.8)	0.837
	Active		3.8 (14.9)		1.1 (15.6)	
Working memory	Placebo	105.7 (12.1)	105.0 (14.1)	0.779	105.6 (11.0)	0.952
	Active	103.5 (13.7)	109.2 (9.3)	0.010	107.7 (10.6)	0.095
∆ Working memory	Placebo		−0.7 (14.3)	0.056	−0.1 (11.6)	0.180
	Active		5.7 (11.6)		4.2 (13.5)	
Sustained attention	Placebo	108.3 (10.0)	108.8 (9.0)	0.767	109.0 (10.3)	0.737
	Active	106.7 (9.9)	110.6 (9.1)	0.020	111.7 (7.2)	0.013
∆ Sustained attention	Placebo		0.6 (10.6)	0.184	0.7 (11.5)	0.126
	Active		3.9 (8.8)		5.0 (10.6)	
Simple attention	Placebo	102.5 (11.6)	103.0 (14.1)	0.892	103.8 (9.8)	0.528
	Active	105.6 (7.3)	81.4 (136.6)	0.337	105.7 (6.8)	0.948
∆ Simple attention	Placebo		0.5 (19.3)	0.320	1.3 (11.3)	0.683
	Active		−24.2 (137.7)		0.1 (10.9)	
Motor speed	Placebo	99.7 (10.7)	101.4 (10.0)	0.101	100.5 (11.4)	0.523
	Active	99.2 (11.7)	100.8 (10.4)	0.129	99.8 (10.2)	0.673
∆ Motor speed	Placebo		1.8 (5.9)	0.926	0.8 (7.1)	0.901
	Active		1.6 (5.8)		0.6 (7.6)	

All data are presented as mean (SD) of Cognitrax scores. Data at week 8 and week 12 were compared with those at week 0 using the paired *t*-test. Comparisons between the placebo and active groups were calculated as changes from week 0, indicated by the “Δ” symbol, and tested using the unpaired *t*-test.

**Table 4:** Post-intervention changes in each quality-of-life parameter (placebo group: *n* = 35; active group: *n* = 32).

		Week 0	Week 8		Week 12	
		Mean (SD)	Mean (SD)	*p*-Value	Mean (SD)	*p*-Value
Physical functioning	Placebo	52.2 (5.4)	51.7 (6.0)	0.682	52.8 (4.8)	0.633
	Active	53.2 (5.0)	53.6 (4.0)	0.660	53.7 (5.1)	0.556
∆ Physical functioning	Placebo		−0.6 (5.4)	0.115	0.6 (4.0)	0.473
	Active		0.4 (3.0)		0.5 (3.1)	
Role physical	Placebo	52.8 (5.6)	53.6 (5.1)	0.534	53.6 (5.5)	0.471
	Active	53.3 (6.8)	53.7 (4.7)	0.873	54 (4.4)	0.710
∆ Role physical	Placebo		0.8 (4.3)	0.895	0.9 (6.0)	0.777
	Active		0.4 (7.2)		0.7 (6.0)	
Bodily pain	Placebo	49.9 (8.8)	48.6 (7.9)	0.472	49.3 (10.1)	0.825
	Active	50.9 (10.3)	50.9 (9.2)	1.000	51.8 (9.4)	0.648
∆ Bodily pain	Placebo		−1.4 (6.8)	0.273	−0.7 (9.6)	0.296
	Active		0.0 (6.2)		0.9 (7.7)	
General health perceptions	Placebo	58.2 (6.9)	57.2 (6.9)	0.256	58.2 (6.8)	1.000
	Active	57.1 (6.8)	57.5 (7.1)	0.846	57.2 (8.0)	0.981
∆ General health perceptions	Placebo		−1.0 (4.3)	0.877	0.0 (4.7)	0.575
	Active		0.4 (3.9)		0.1 (5.2)	
Vitality	Placebo	55.5 (7.0)	55.8 (7.7)	0.930	56.0 (6.2)	0.854
	Active	56.8 (6.7)	57.2 (6.2)	0.862	57.0 (6.4)	0.963
∆ Vitality	Placebo		0.3 (6.6)	0.443	0.5 (6.5)	0.535
	Active		0.4 (5.0)		0.2 (5.6)	
Social functioning	Placebo	54.2 (6.6)	55.3 (6.3)	0.423	55.0 (6.3)	0.632
	Active	53.5 (7.0)	55.1 (5.7)	0.183	55.2 (5.2)	0.128
∆ Social functioning	Placebo		1.1 (5.1)	0.877	0.8 (7.0)	0.850
	Active		1.6 (6.7)		1.8 (5.6)	
Role emotional	Placebo	52.8 (6.3)	54.9 (4.5)	0.041	54.6 (4.3)	0.093
	Active	54.4 (5.1)	53.8 (4.8)	0.779	54.9 (4.2)	0.783
∆ Role emotional	Placebo		2.1 (6.1)	0.332	1.8 (6.3)	0.720
	Active		−0.6 (7.2)		0.6 (3.2)	
Mental health	Placebo	56.9 (4.4)	57.6 (5.5)	0.663	57.6 (6.9)	0.718
	Active	57.1 (6.7)	57.1 (6.5)	0.993	57.5 (5.4)	0.775
∆ Mental health	Placebo		0.7 (6.1)	0.732	0.7 (5.2)	0.982
	Active		0.1 (5.1)		0.5 (4.4)	

All data are presented as mean (SD) of SF-36v2^®^ scores. Data at week 8 and week 12 were compared with those at week 0 using Dunnett’s test. Comparisons between the placebo and active groups were calculated as changes from week 0, indicated by the “Δ” symbol, and tested using the unpaired *t*-test.

**Table 5:** Post-intervention changes in each cognitive function parameter (placebo group: *n* = 32; active group: *n* = 31).

Parameter		Week 0	Week 12		Change	
		Mean (SD)	Mean (SD)	*p*-Value	Mean (SD)	*p*-Value
LIF-R, NPX	Placebo	3.36 (0.27)	3.39 (0.23)	0.459	0.02 (0.19)	0.012
	Active	3.49 (0.30)	3.40 (0.20)	0.008	−0.10 (0.19)	
ST1A1, NPX	Placebo	2.19 (1.16)	1.75 (0.83)	0.111	−0.36 (1.10)	0.020
	Active	1.82 (0.82)	2.10 (1.00)	0.079	0.33 (0.86)	
CCL23, NPX	Placebo	10.86 (0.35)	10.88 (0.25)	0.718	0.02 (0.25)	0.021
	Active	11.09 (0.46)	10.90 (0.40)	0.012	−0.15 (0.32)	
TWEAK, NPX	Placebo	9.10 (0.34)	9.08 (0.33)	0.658	−0.01 (0.18)	0.033
	Active	9.22 (0.26)	9.10 (0.20)	0.002	−0.12 (0.19)	
ADA, NPX	Placebo	5.39 (0.41)	5.46 (0.39)	0.068	0.08 (0.23)	0.052
	Active	5.53 (0.37)	5.50 (0.30)	0.345	−0.04 (0.24)	
OPG, NPX	Placebo	10.49 (0.4)	10.58 (0.41)	0.134	0.09 (0.34)	0.052
	Active	10.60 (0.30)	10.60 (0.20)	0.188	−0.04 (0.17)	
CCL11, NPX	Placebo	8.66 (0.43)	8.76 (0.36)	0.029	0.09 (0.24)	0.054
	Active	8.72 (0.29)	8.70 (0.20)	0.649	−0.02 (0.21)	
GDNF, NPX	Placebo	1.98 (0.47)	2.19 (0.44)	0.003	0.20 (0.33)	0.055
	Active	2.15 (0.33)	2.10 (0.30)	0.447	0.04 (0.28)	
CX3CL1, NPX	Placebo	3.66 (0.48)	3.82 (0.51)	0.059	0.17 (0.48)	0.058
	Active	3.86 (0.41)	3.80 (0.40)	0.520	−0.04 (0.34)	
IL-8, NPX	Placebo	5.97 (0.41)	6.22 (0.49)	0.012	0.24 (0.53)	0.063
	Active	6.15 (0.75)	6.20 (0.70)	0.733	0.02 (0.37)	
CCL28, NPX	Placebo	2.62 (0.50)	2.64 (0.49)	0.711	0.02 (0.27)	0.063
	Active	2.73 (0.46)	2.60 (0.50)	0.009	−0.09 (0.18)	
IL-18, NPX	Placebo	9.10 (0.67)	9.28 (0.68)	0.002	0.18 (0.30)	0.064
	Active	9.14 (0.63)	9.20 (0.50)	0.912	0.01 (0.41)	
IL-10, NPX	Placebo	3.22 (0.52)	3.44 (0.78)	0.035	0.22 (0.56)	0.064
	Active	3.47 (0.44)	3.50 (0.40)	0.975	0.00 (0.28)	
CCL19, NPX	Placebo	8.73 (0.77)	8.88 (0.90)	0.163	0.15 (0.60)	0.081
	Active	8.72 (0.49)	8.70 (0.60)	0.278	−0.06 (0.31)	
CD5, NPX	Placebo	5.92 (0.40)	5.91 (0.37)	0.864	−0.01 (0.19)	0.092
	Active	6.02 (0.39)	5.90 (0.30)	0.017	−0.09 (0.20)	

All data were obtained using Olink^®^ Target 96 Inflammation Panels and are presented as the mean (SD) of log base 2-normalized protein expression values (NPX). Data at week 12 were compared with those at week 0 using the paired *t*-test. Comparisons between the placebo and active groups were evaluated by calculating the change in measurements at week 0 and 12 in both groups using unpaired *t*-tests. LIF-R, leukemia inhibitory factor receptor; ST1A1, sulfotransferase 1A1; CCL23, C-C motif hemokine 23; TWEAK, tumor necrosis factor (ligand) superfamily, member 12; ADA, adenosine deaminase; OPG, osteoprotegerin; CCL11, eotaxin; GDNF, glial cell line-derived neurotrophic factor; CX3CL1, fractalkine; IL-8, interleukin 8; CCL28, C-C motif chemokine 28; IL-18, interleukin 18; IL-10, interleukin 10; CCL19, C-C motif chemokine 19; CD5, T-cell surface glycoprotein CD5.

## Data Availability

Datasets generated during the current study and/or analyzed during the current study are available from the responsible author upon reasonable request.

## Supplementary Materials

The following supporting information can be downloaded at: https://www.mdpi.com/article/10.3390/nu15194175/s1, Table S1: CONSORT 2010 checklist of information to include when reporting a randomized trial. Table S2: Post-intervention changes in Cognitrax scores (placebo group: *n* = 32; active group: *n* = 31), Table S3: Post-intervention changes in each inflammatory marker (all data), Table S4: Post-intervention changes in IL-1β and *hs*-CRP scores, Figure S1: Principal coordinate analysis with *t*-distribution ellipses between the active and the placebo groups.
