# Supplementary material for: Effect of Continuous Ingestion of Bifidobacteria and Dietary Fiber on Improvement in Cognitive Function: A Randomized, Double-Blind, Placebo-Controlled Trial
Source: Nutrients. 2023 Sep 27;15(19):4175. doi: 10.3390/nu15194175 (PMC10574581; doi:10.3390/nu15194175)
Supplement: Supplementary file 1 [file nutrients-15-04175-s001.zip › Supplementary materials/Supplementary Figure S1.pdf]

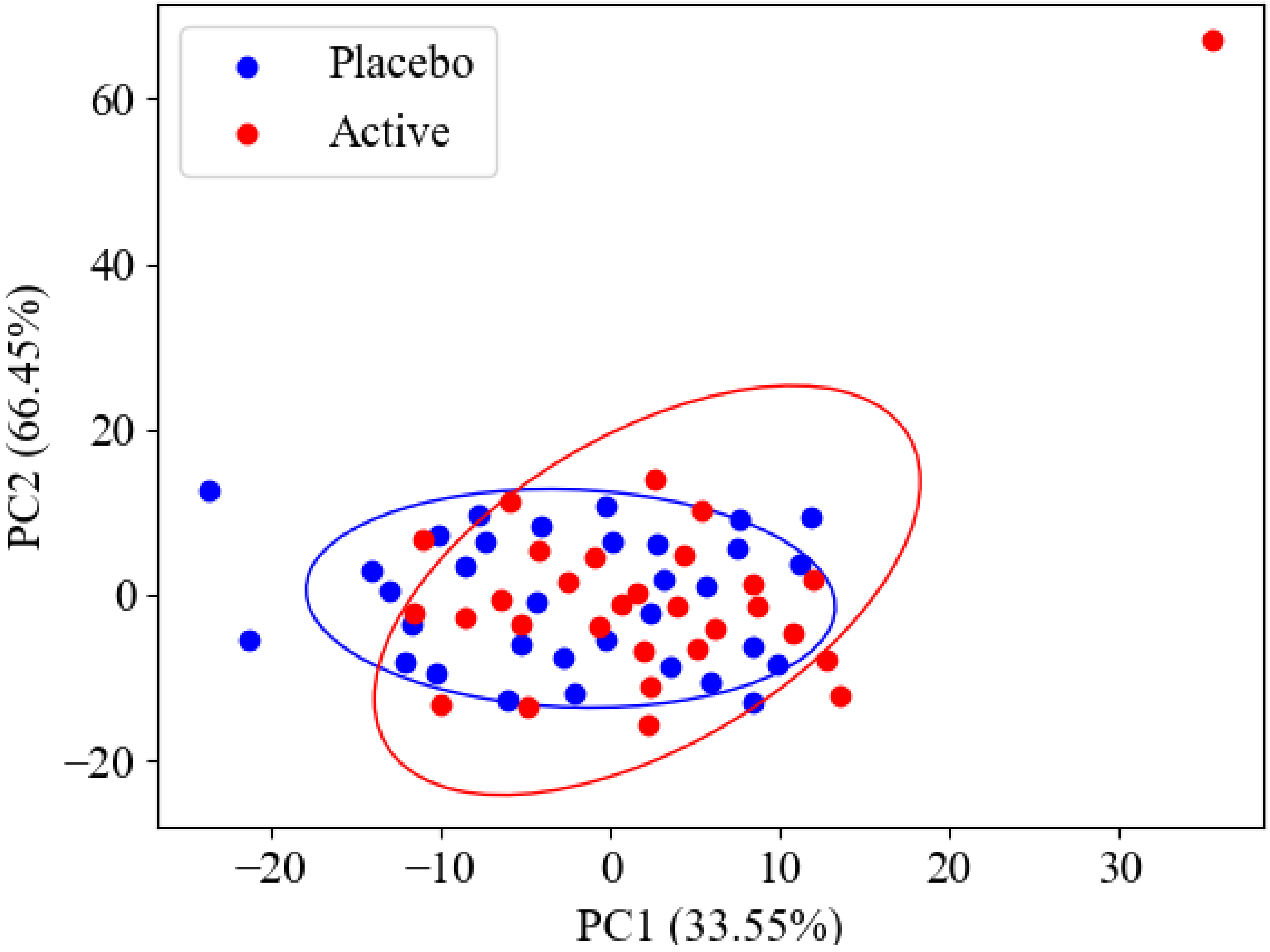

Figure S1. Principal coordinate analysis with *t*-distribution ellipses between the placebo and the active groups.
