# Supplementary material for: Effect of Continuous Ingestion of Bifidobacteria and Dietary Fiber on Improvement in Cognitive Function: A Randomized, Double-Blind, Placebo-Controlled Trial
Source: Nutrients. 2023 Sep 27;15(19):4175. doi: 10.3390/nu15194175 (PMC10574581; doi:10.3390/nu15194175)
Supplement: Supplementary file 1 [file nutrients-15-04175-s001.zip › Supplementary materials/Supplementary Table S2.pdf]

Table S2. Post-intervention changes in Cognitrax scores (placebo group: n = 32; active group: n = 31).

Task			Week 0	Week 12		Change	
			Mean (SD)	Mean (SD)	P−value	Mean (SD)	P−value
VBM	Correct hits (immediate)	Placebo	102.8 (13.5)	106.2 (12.4)	0.119	103.8 (13.6)	0.688
		Active	103.5 (11.7)	107.9 (10.6)	0.047	107.7 (11.6)	0.068
		Placebo		3.5 (12.2)	0.763	1.0 (14.0)	0.344
		Active		4.4 (11.8)		4.2 (12.2)	
	Correct passes (immediate)	Placebo	107.3 (7.5)	106.2 (9.1)	0.498	105.9 (7.0)	0.506
		Active	103.5 (19.0)	102.6 (13.2)	0.762	106.1 (10.2)	0.387
		Placebo		−1.1 (9.0)	0.936	−1.3 (11.0)	0.271
		Active		−0.8 (15.3)		2.6 (16.4)	
	Correct hits (delayed)	Placebo	103.5 (12.4)	101.1 (15.6)	0.319	101.6 (13.7)	0.447
		Active	100.9 (13.6)	103.8 (16.2)	0.315	105.4 (15.2)	0.148
		Placebo		−2.4 (13.6)	0.156	−1.9 (14.0)	0.106
		Active		2.9 (15.8)		4.5 (16.8)	
	Correct passes (delayed)	Placebo	102.0 (11.9)	104.4 (8.9)	0.338	103.7 (10.3)	0.541
		Active	105.6 (8.5)	105.2 (9.2)	0.727	102.4 (12.4)	0.169
		Placebo		2.3 (13.6)	0.311	1.7 (15.2)	0.170
		Active		−0.5 (7.1)		−3.3 (13.0)	
VIM	Correct hits (immediate)	Placebo	95.2 (15.8)	93.8 (17.2)	0.698	90.8 (16.5)	0.236
		Active	95.0 (14.8)	99.4 (13.4)	0.170	96.6 (10.9)	0.574
		Placebo		−1.3 (19.0)	0.217	−4.3 (20.3)	0.202
		Active		4.5 (17.6)		1.7 (16.4)	

	Correct passes (immediate)	Placebo	109.3 (10.8)	107.4 (9.5)	0.314	108.3 (12.3)	0.587
		Active	108.4 (9.9)	108.8 (10.1)	0.867	109.2 (12.8)	0.733
		Placebo		−1.9 (10.5)	0.455	−1.1 (10.9)	0.542
		Active		0.4 (13.9)		0.8 (13.5)	
	Correct hits (delayed)	Placebo	92.5 (15.7)	88.9 (17.8)	0.232	95.1 (17.2)	0.401
		Active	96.8 (13.9)	99.3 (14.9)	0.267	96.5 (15.2)	0.909
		Placebo		−3.6 (16.5)	0.105	2.6 (17.4)	0.469
		Active		2.5 (12.1)		−0.3 (14.1)	
	Correct passes (delayed)	Placebo	109.2 (11.6)	105.1 (14.8)	0.205	102.3 (14.4)	0.014
		Active	106.0 (12.9)	103.2 (13.2)	0.186	104.5 (15.0)	0.497
		Placebo		−4.1 (17.8)	0.748	−6.9 (14.9)	0.120
		Active		−2.8 (11.7)		−1.5 (12.0)	
FTT	Right taps, average	Placebo	102.0 (9.0)	103.6 (8.8)	0.153	103.1 (11.2)	0.441
		Active	101.6 (12.3)	103.5 (10.1)	0.074	102.8 (10.9)	0.350
		Placebo		1.6 (6.3)	0.887	1.2 (8.4)	0.985
		Active		1.8 (5.5)		1.2 (7.0)	
	Left taps, average	Placebo	97.3 (12.2)	99.0 (11.3)	0.130	97.8 (12.0)	0.673
		Active	96.5 (11.2)	97.8 (11.2)	0.309	96.5 (10.9)	0.984
		Placebo		1.7 (6.1)	0.811	0.5 (6.6)	0.788
		Active		1.3 (6.9)		0.0 (8.8)	
SDC	Correct responses	Placebo	113.5 (8.4)	115.1 (9.5)	0.199	116.2 (9.5)	0.028
		Active	113.5 (8.5)	116.8 (11.0)	0.031	117.5 (9.2)	0.004

		Placebo		1.6 (6.7)	0.354	2.7 (6.7)	0.477
		Active		3.3 (8.2)		4.0 (7.2)	
	Errors	Placebo	106.8 (8.4)	107.0 (9.9)	0.926	104.1 (12.7)	0.303
		Active	102.9 (12.5)	106.8 (7.2)	0.193	107.8 (7.6)	0.092
		Placebo		0.2 (13.2)	0.332	-2.7 (14.5)	0.051
		Active		3.8 (16.1)		4.9 (15.6)	
ST (part 1)	Simple reaction time (ms)	Placebo	93.7 (11.0)	97.5 (6.7)	0.025	98.4 (7.0)	0.009
		Active	95.9 (9.7)	97.0 (9.7)	0.551	98.3 (9.6)	0.191
		Placebo		3.8 (9.2)	0.252	4.8 (9.6)	0.330
		Active		1.1 (9.8)		2.4 (9.8)	
ST (part 2)	Complex reaction time, correct (ms)	Placebo	93.0 (11.2)	93.6 (11.1)	0.695	96.2 (10.4)	0.018
		Active	95.5 (10.1)	96.7 (11.6)	0.419	99.2 (10.2)	0.011
		Placebo		0.6 (8.0)	0.740	3.2 (7.1)	0.767
		Active		1.3 (8.5)		3.7 (7.6)	
ST (part 3)	Stroop reaction time, correct (ms)	Placebo	96.9 (10.6)	96.8 (12.1)	0.896	101.1 (10.9)	<0.001
		Active	98.3 (8.5)	100.8 (8.8)	0.046	102.4 (8.3)	0.008
		Placebo		−0.2 (6.7)	0.119	4.2 (4.8)	0.957
		Active		2.5 (6.7)		4.1 (8.0)	
	Stroop commission errors	Placebo	99.5 (12.7)	104.9 (6.4)	0.015	102.0 (8.9)	0.228
		Active	99.6 (9.2)	101.8 (10.4)	0.405	102.1 (7.4)	0.192
		Placebo		5.5 (12.0)	0.322	2.5 (11.5)	0.977
		Active		2.2 (14.3)		2.4 (10.1)	

SAT	Correct responses	Placebo	98.9 (9.6)	101.5 (8.6)	0.010	103.9 (10.0)	<0.001
		Active	93.4 (10.4)	98.2 (10.5)	0.020	102.7 (7.4)	<0.001
		Placebo		2.6 (5.4)	0.318	5.1 (7.2)	0.064
		Active		4.8 (11.0)		9.3 (10.5)	
	Errors	Placebo	108.7 (8.5)	110.5 (5.0)	0.117	111.3 (6.9)	0.023
		Active	102.8 (11.8)	107.9 (7.4)	0.032	111.4 (4.9)	<0.001
		Placebo		1.8 (6.5)	0.200	2.6 (6.1)	0.017
		Active		5.1 (12.6)		8.6 (12.1)	
	Reaction time, correct (ms)	Placebo	102.0 (12.1)	104.4 (11.7)	0.100	108.0 (11.8)	0.001
		Active	100.1 (10.1)	101.9 (10.6)	0.383	106.5 (8.1)	0.001
		Placebo		2.4 (8.0)	0.798	6.0 (9.8)	0.906
		Active		1.8 (11.2)		6.3 (9.7)	
CPT	Correct responses	Placebo	93.8 (32.0)	97.4 (31.2)	0.657	98.8 (15.3)	0.426
		Active	101.6 (8.5)	82.5 (112.0)	0.356	99.9 (12.8)	0.552
		Placebo		3.6 (45.8)	0.298	5.0 (35.1)	0.334
		Active		−19.0 (112.9)		−1.7 (15.8)	
	Omission errors	Placebo	93.8 (32.0)	97.4 (31.2)	0.657	98.8 (15.3)	0.426
		Active	101.6 (8.5)	82.5 (112.0)	0.356	99.9 (12.8)	0.552
		Placebo		3.6 (45.8)	0.298	5.0 (35.1)	0.334
		Active		−19.0 (112.9)		−1.7 (15.8)	
	Commission errors	Placebo	105.4 (6.6)	104.0 (10.0)	0.518	104.8 (8.5)	0.655
		Active	106.4 (5.4)	86.8 (105.2)	0.315	106.7 (5.2)	0.837

		Placebo		−1.4 (12.2)	0.343	−0.6 (7.1)	0.658
		Active		−19.5 (106.4)		0.3 (8.7)	
	Reaction time, correct	Placebo	85.0 (17.5)	86.8 (14.1)	0.370	85.8 (15.2)	0.574
		Active	90.9 (10.5)	90.9 (12.3)	0.958	90.8 (11.2)	0.929
		Placebo		1.9 (11.7)	0.199	0.8 (8.1)	0.615
		Active		−2.9 (17.3)		−0.1 (6.0)	
POET	Correct responses	Placebo	85.3 (19.2)	88.9 (17.1)	0.305	84.7 (19.3)	0.864
		Active	81.2 (24.7)	92.2 (19.6)	0.013	95.5 (15.7)	0.002
		Placebo		3.6 (19.7)	0.178	−0.6 (19.5)	0.007
		Active		11.0 (23.2)		14.3 (22.8)	
	Average reaction time, correct (ms)	Placebo	91.2 (17.6)	87.3 (17.7)	0.062	92.3 (19.6)	0.564
		Active	90.6 (16.6)	91.5 (18.8)	0.756	94.0 (18.5)	0.216
		Placebo		−3.9 (11.4)	0.167	1.1 (10.9)	0.494
		Active		0.9 (15.5)		3.4 (14.9)	
	Omission errors	Placebo	85.3 (19.2)	88.9 (17.1)	0.305	84.7 (19.3)	0.864
		Active	81.2 (24.7)	92.2 (19.6)	0.013	95.5 (15.7)	0.002
		Placebo		3.6 (19.7)	0.178	−0.6 (19.5)	0.007
		Active		11.0 (23.2)		14.3 (22.8)	
	Commission errors	Placebo	92.8 (16.3)	97.1 (16.3)	0.095	101.0 (16.8)	0.002
		Active	98.4 (16.0)	98.2 (15.9)	0.939	100.1 (14.6)	0.597
		Placebo		4.3 (14.1)	0.243	8.3 (13.9)	0.109
		Active		−0.2 (16.2)		1.7 (17.8)	

POET	Correct hits	Placebo	98.4 (12.6)	96.2 (14.8)	0.475	90.3 (24.0)	0.055
(positive emotion)		Active	89.5 (21.1)	100.6 (11.4)	0.003	97.2 (17.3)	0.020
		Placebo		−2.2 (16.9)	0.005	−8.1 (22.9)	0.003
		Active		11.1 (19.2)		7.7 (17.5)	
	Reaction time (ms)	Placebo	80.6 (21.7)	77.3 (22.3)	0.244	82.3 (23.0)	0.522
		Active	81.2 (19.9)	82.9 (19.9)	0.628	85.2 (22.0)	0.244
		Placebo		−3.3 (15.8)	0.262	1.8 (15.3)	0.603
		Active		1.7 (19.1)		4.0 (18.8)	
POET	Correct hits	Placebo	78.6 (25.9)	85.8 (27.8)	0.146	86.4 (22.6)	0.084
(negative emotion)		Active	79.0 (33.7)	85.2 (27.3)	0.284	93.9 (24.0)	0.006
		Placebo		7.2 (27.4)	0.891	7.8 (24.6)	0.293
		Active		6.2 (31.6)		14.8 (28.1)	
	Reaction time (ms)	Placebo	100.3 (14.3)	96.6 (15.4)	0.146	101.0 (16.5)	0.673
		Active	98.5 (14.7)	99.0 (18.6)	0.860	101.1 (15.1)	0.275
		Placebo		−3.7 (13.9)	0.262	0.7 (9.5)	0.517
		Active		0.5 (15.2)		2.6 (12.9)	
NVRT	Correct responses	Placebo	98.2 (15.2)	95.7 (16.4)	0.386	98.8 (15.3)	0.822
		Active	96.1 (14.5)	99.8 (12.4)	0.160	98.0 (12.8)	0.488
		Placebo		−2.5 (15.9)	0.111	0.6 (14.8)	0.738
		Active		3.7 (14.3)		1.8 (14.6)	
	Average reaction time, correct (ms)	Placebo	113.2 (11.5)	113.4 (11.9)	0.945	113.8 (9.0)	0.784
		Active	112.5 (8.3)	114.2 (10.0)	0.441	116.0 (6.9)	0.037

		Placebo		0.2 (12.7)	0.631	0.6 (12.2)	0.287
		Active		1.6 (11.7)		3.5 (8.9)	
	Commission errors	Placebo	92.1 (16.5)	88.5 (17.8)	0.222	92.2 (18.2)	0.973
		Active	90.8 (16.3)	94.0 (15.3)	0.223	91.0 (14.1)	0.963
		Placebo		−3.6 (16.4)	0.084	0.1 (15.6)	0.993
		Active		3.2 (14.3)		0.1 (15.2)	
	Omission errors	Placebo	108.9 (9.4)	110.7 (7.2)	0.322	109.3 (10.5)	0.812
		Active	107.5 (11.1)	108.7 (9.6)	0.517	110.5 (8.2)	0.063
		Placebo		1.8 (10.2)	0.810	0.4 (9.6)	0.264
		Active		1.2 (10.1)		3.0 (8.6)	
FPCPT (part 3)	Correct responses	Placebo	111.9 (12.8)	111.3 (6.9)	0.802	112.0 (13.4)	0.975
		Active	111.0 (10.7)	113.2 (20.5)	0.549	116.5 (8.4)	0.007
		Placebo		−0.6 (14.0)	0.520	0.1 (16.5)	0.126
		Active		2.2 (20.5)		5.5 (10.7)	
	Average response time, correct (ms)	Placebo	100.5 (15.7)	99.9 (14.6)	0.834	99.5 (12.7)	0.632
		Active	103.5 (9.0)	100.6 (17.9)	0.383	104.1 (8.0)	0.680
		Placebo		-0.5 (14.2)	0.564	−1.0 (11.3)	0.534
		Active		−2.9 (18.4)		0.5 (6.9)	
	Incorrect responses	Placebo	102.2 (12.1)	102.1 (11.5)	0.984	101.1 (14.5)	0.751
		Active	103.8 (2.5)	100.5 (15.4)	0.196	103.2 (3.0)	0.275
		Placebo		−0.1 (17.1)	0.414	−1.1 (19.4)	0.899
		Active		−3.3 (13.9)		-0.6 (3.2)	

	Average response time, incorrect (ms)	Placebo	73.0 (−)	76.0 (9.9)	−	62.0 (16.3)	−
		Active	77.3 (12.4)	77.3 (7.6)	0.395	80.3 (15.7)	0.979
		Placebo		− (−)	−	− (−)	−
		Active		7.0 (7.1)		−1.0 (42.4)	
	Omission errors	Placebo	111.9 (12.8)	111.3 (6.9)	0.802	112.0 (13.4)	0.975
		Active	111.0 (10.7)	113.2 (20.5)	0.549	116.5 (8.4)	0.007
		Placebo		−0.6 (14.0)	0.520	0.1 (16.5)	0.126
		Active		2.2 (20.5)		5.5 (10.7)	
FPCPT (part 4)	Correct responses	Placebo	106.7 (14.9)	105.4 (16.2)	0.698	108.2 (12.2)	0.535
		Active	105.3 (13.8)	112.4 (10.1)	<0.001	108.9 (11.8)	0.130
		Placebo		−1.3 (19.0)	0.028	1.5 (13.8)	0.530
		Active		7.2 (9.1)		3.7 (13.1)	
	Average response time, correct (ms)	Placebo	101.1 (13.6)	103.8 (9.0)	0.219	103.1 (11.4)	0.315
		Active	103.2 (8.9)	104.9 (8.3)	0.249	104.8 (11.9)	0.380
		Placebo		2.8 (12.4)	0.683	2.1 (11.4)	0.859
		Active		1.7 (7.9)		1.6 (9.9)	
	Incorrect responses	Placebo	100.5 (11.8)	101.5 (11.4)	0.677	98.0 (13.0)	0.296
		Active	98.7 (12.3)	98.7 (11.0)	1.000	101.1 (7.9)	0.267
		Placebo		1.1 (14.3)	0.768	−2.5 (13.3)	0.128
		Active		0.0 (14.1)		2.4 (11.6)	
	Average response time, incorrect (ms)	Placebo	95.9 (4.8)	95.0 (5.8)	0.747	93.5 (8.4)	0.195
		Active	97.5 (5.2)	94.9 (7.8)	0.313	95.5 (6.6)	0.392

	Placebo		0.6 (7.3)	0.351	−3.4 (10.2)	0.491
	Active		−1.9 (7.4)		−1.4 (7.2)	
Omission errors	Placebo	106.7 (14.9)	105.4 (16.2)	0.698	108.2 (12.2)	0.535
	Active	105.3 (13.8)	112.4 (10.1)	<0.001	108.9 (11.8)	0.130
	Placebo		−1.3 (19.0)	0.028	1.5 (13.8)	0.530
	Active		7.2 (9.1)		3.7 (13.1)	

Data at week 12 were compared with those at week 0, using the paired *t*-test.

Comparisons between the placebo and active groups were tested by the unpaired *t*-test.

VBM, verbal memory test; VIM, visual memory test; FTT, finger tapping test; SDC, symbol digit coding test; ST, Stroop test; SAT, shift attention test; CPT, continuous performance test; POET, perception of emotion test; NVRT, nonverbal reasoning test; FPCPT, four-part continuous performance test
