# Supplementary material for: Effect of Continuous Ingestion of Bifidobacteria and Dietary Fiber on Improvement in Cognitive Function: A Randomized, Double-Blind, Placebo-Controlled Trial
Source: Nutrients. 2023 Sep 27;15(19):4175. doi: 10.3390/nu15194175 (PMC10574581; doi:10.3390/nu15194175)
Supplement: Supplementary file 1 [file nutrients-15-04175-s001.zip › Supplementary materials/Supplementary Table S3.pdf]

Table S3. Post-intervention changes in each inflammatory marker (all data).

		Week 0	Week 12		Change	
		Mean (SD)	Mean (SD)	*P*−value	Mean (SD)	*P*−value
Adenosine deaminase (ADA)	Placebo	5.39 (0.41)	5.46 (0.39)	0.068	0.08 (0.23)	0.052
	Active	5.53 (0.37)	5.50 (0.30)	0.345	−0.04 (0.24)	
Axin-1 (AXIN1)	Placebo	1.46 (0.59)	1.63 (0.47)	0.149	0.18 (0.66)	0.910
	Active	1.50 (0.55)	1.70 (0.60)	0.101	0.20 (0.61)	
Caspase-8 (CASP-8)	Placebo	1.48 (0.43)	1.60 (0.44)	0.065	0.11 (0.33)	0.786
	Active	1.63 (0.39)	1.70 (0.40)	0.052	0.09 (0.25)	
C-C motif chemokine 19 (CCL19)	Placebo	8.73 (0.77)	8.88 (0.90)	0.163	0.15 (0.60)	0.081
	Active	8.72 (0.49)	8.70 (0.60)	0.278	−0.06 (0.31)	
C-C motif chemokine 20 (CCL20)	Placebo	7.60 (0.91)	7.75 (1.19)	0.288	0.15 (0.82)	0.121
	Active	7.30 (0.66)	7.20 (0.60)	0.215	−0.11 (0.46)	
C-C motif chemokine 23 (CCL23)	Placebo	10.86 (0.35)	10.88 (0.25)	0.718	0.02 (0.25)	0.021
	Active	11.09 (0.46)	10.90 (0.40)	0.012	−0.15 (0.32)	
C-C motif chemokine 25 (CCL25)	Placebo	6.72 (0.64)	6.83 (0.60)	0.090	0.10 (0.34)	0.203
	Active	6.83 (0.55)	6.80 (0.50)	0.944	0.00 (0.27)	
C-C motif chemokine 28 (CCL28)	Placebo	2.62 (0.50)	2.64 (0.49)	0.711	0.02 (0.27)	0.063
	Active	2.73 (0.46)	2.60 (0.50)	0.009	−0.09 (0.18)	
C-C motif chemokine 3 (CCL3)	Placebo	7.20 (0.68)	7.35 (1.02)	0.152	0.15 (0.59)	0.624
	Active	7.17 (0.72)	7.30 (0.80)	0.164	0.09 (0.34)	
C-C motif chemokine 4 (CCL4)	Placebo	8.07 (0.56)	8.26 (0.93)	0.118	0.18 (0.65)	0.354
	Active	8.21 (0.60)	8.30 (0.60)	0.387	0.06 (0.36)	

CD40L receptor (CD40)	Placebo	11.28 (0.48)	11.27 (0.38)	0.828	−0.01 (0.31)	0.816
	Active	11.38 (0.42)	11.30 (0.40)	0.565	−0.03 (0.27)	
CUB domain-containing protein 1 (CDCP1)	Placebo	2.44 (0.65)	2.49 (0.60)	0.347	0.05 (0.28)	0.227
	Active	2.54 (0.72)	2.50 (0.60)	0.442	−0.04 (0.28)	
C-X-C motif chemokine 1 (CXCL1)	Placebo	9.66 (0.49)	9.72 (0.48)	0.294	0.05 (0.29)	0.688
	Active	9.79 (0.58)	9.80 (0.60)	0.724	0.02 (0.34)	
C-X-C motif chemokine 10 (CXCL10)	Placebo	9.51 (0.84)	9.71 (1.12)	0.187	0.20 (0.84)	0.394
	Active	9.38 (0.56)	9.40 (0.70)	0.725	0.04 (0.59)	
C-X-C motif chemokine 11 (CXCL11)	Placebo	8.31 (0.59)	8.54 (0.92)	0.148	0.24 (0.92)	0.340
	Active	8.38 (0.52)	8.40 (0.60)	0.430	0.06 (0.41)	
C-X-C motif chemokine 5 (CXCL5)	Placebo	11.74 (0.56)	11.77 (0.51)	0.499	0.03 (0.29)	0.286
	Active	11.83 (0.61)	11.80 (0.60)	0.413	−0.05 (0.30)	
C-X-C motif chemokine 6 (CXCL6)	Placebo	9.23 (0.61)	9.22 (0.57)	0.824	−0.01 (0.28)	0.594
	Active	9.35 (0.46)	9.30 (0.40)	0.267	−0.04 (0.21)	
C-X-C motif chemokine 9 (CXCL9)	Placebo	7.18 (0.74)	7.30 (0.84)	0.156	0.12 (0.47)	0.442
	Active	7.02 (0.60)	7.00 (0.80)	0.950	0.01 (0.66)	
Cystatin D (CST5)	Placebo	5.79 (0.61)	5.84 (0.63)	0.330	0.04 (0.25)	0.870
	Active	5.95 (0.62)	6.00 (0.60)	0.448	0.03 (0.24)	
Delta and Notch-like epidermal growth factor-related receptor (DNER)	Placebo	9.11 (0.34)	9.06 (0.26)	0.226	−0.05 (0.25)	0.280
	Active	9.20 (0.30)	9.10 (0.30)	0.003	−0.12 (0.20)	
Eotaxin (CCL11)	Placebo	8.66 (0.43)	8.76 (0.36)	0.029	0.09 (0.24)	0.054
	Active	8.72 (0.29)	8.70 (0.20)	0.649	−0.02 (0.21)	

Eukaryotic translation initiation factor 4E-binding protein 1 (4E-BP1)	Placebo	7.32 (0.70)	7.55 (0.69)	0.150	0.23 (0.90)	0.811
	Active	7.34 (0.67)	7.50 (0.80)	0.328	0.17 (0.96)	
Fibroblast growth factor 19 (FGF-19)	Placebo	9.19 (0.91)	9.44 (0.84)	0.135	0.25 (0.94)	0.337
	Active	9.11 (0.90)	9.10 (1.00)	0.865	0.03 (0.88)	
Fibroblast growth factor 21 (FGF-21)	Placebo	5.84 (1.26)	6.00 (1.56)	0.286	0.17 (0.88)	0.273
	Active	5.75 (1.23)	5.60 (1.00)	0.633	−0.07 (0.81)	
Fibroblast growth factor 5 (FGF-5)	Placebo	2.42 (0.29)	2.47 (0.25)	0.094	0.05 (0.17)	0.180
	Active	2.59 (0.28)	2.60 (0.20)	0.723	−0.01 (0.21)	
Fms-related tyrosine kinase 3 ligand (Flt3L)	Placebo	9.37 (0.45)	9.39 (0.45)	0.634	0.03 (0.31)	0.854
	Active	9.29 (0.49)	9.30 (0.40)	0.795	0.01 (0.26)	
Fractalkine (CX3CL1)	Placebo	3.66 (0.48)	3.82 (0.51)	0.059	0.17 (0.48)	0.058
	Active	3.86 (0.41)	3.80 (0.40)	0.520	−0.04 (0.34)	
Glial cell line-derived neurotrophic factor (GDNF)	Placebo	1.98 (0.47)	2.19 (0.44)	0.003	0.20 (0.33)	0.055
	Active	2.15 (0.33)	2.10 (0.30)	0.447	0.04 (0.28)	
Hepatocyte growth factor (HGF)	Placebo	9.64 (0.45)	9.76 (0.47)	0.010	0.13 (0.27)	0.282
	Active	9.66 (0.48)	9.70 (0.40)	0.502	0.04 (0.35)	
Interferon gamma (IFN-gamma)	Placebo	6.57 (0.69)	6.75 (0.97)	0.226	0.17 (0.81)	0.166
	Active	6.72 (0.69)	6.60 (0.70)	0.470	−0.08 (0.63)	
Interleukin-6 (IL-6)	Placebo	2.72 (0.58)	2.94 (0.78)	0.031	0.22 (0.55)	0.623
	Active	2.93 (0.74)	3.10 (0.80)	0.491	0.12 (0.95)	
Interleukin-7 (IL-7)	Placebo	4.43 (0.44)	4.61 (0.44)	0.009	0.18 (0.37)	0.127
	Active	4.52 (0.47)	4.50 (0.50)	0.739	0.03 (0.41)	

Interleukin-8 (IL-8)	Placebo	5.97 (0.41)	6.22 (0.49)	0.012	0.24 (0.53)	0.063
	Active	6.15 (0.75)	6.20 (0.70)	0.733	0.02 (0.37)	
Interleukin-10 (IL-10)	Placebo	3.22 (0.52)	3.44 (0.78)	0.035	0.22 (0.56)	0.064
	Active	3.47 (0.44)	3.50 (0.40)	0.975	0.00 (0.28)	
Interleukin-10 receptor subunit alpha (IL-10RA)	Placebo	1.65 (0.91)	1.78 (0.98)	0.028	0.11 (0.27)	0.151
	Active	1.78 (0.75)	1.80 (0.80)	0.806	0.01 (0.25)	
Interleukin-10 receptor subunit beta (IL-10RB)	Placebo	7.18 (0.35)	7.20 (0.30)	0.418	0.02 (0.16)	0.178
	Active	7.30 (0.30)	7.30 (0.30)	0.274	−0.03 (0.15)	
Interleukin-12 subunit beta (IL-12B)	Placebo	6.78 (0.69)	6.78 (0.75)	0.968	0.00 (0.33)	0.269
	Active	6.86 (0.37)	6.80 (0.40)	0.069	−0.08 (0.25)	
Interleukin-15 receptor subunit alpha (IL-15RA)	Placebo	1.56 (0.44)	1.66 (0.40)	0.022	0.10 (0.22)	0.333
	Active	1.67 (0.36)	1.70 (0.30)	0.181	0.05 (0.18)	
Interleukin-17A (IL-17A)	Placebo	1.77 (0.67)	1.79 (0.62)	0.607	−0.05 (0.50)	0.724
	Active	1.96 (0.76)	1.90 (0.70)	0.080	−0.09 (0.24)	
Interleukin-17C (IL-17C)	Placebo	2.95 (0.89)	2.83 (0.97)	0.537	−0.12 (1.07)	0.718
	Active	2.91 (0.54)	2.70 (0.50)	0.032	−0.19 (0.47)	
Interleukin-18 (IL-18)	Placebo	9.10 (0.67)	9.28 (0.68)	0.002	0.18 (0.30)	0.064
	Active	9.14 (0.63)	9.20 (0.50)	0.912	0.01 (0.41)	
Interleukin-18 receptor 1 (IL-18R1)	Placebo	8.23 (0.55)	8.37 (0.53)	0.006	0.14 (0.27)	0.214
	Active	8.29 (0.36)	8.40 (0.40)	0.115	0.06 (0.21)	
Latency-associated peptide transforming growth factor beta-1 (LAP TGF-beta-1)	Placebo	8.51 (0.36)	8.42 (0.37)	0.255	−0.09 (0.44)	0.933
	Active	8.59 (0.37)	8.50 (0.40)	0.223	−0.10 (0.43)	

Leukemia inhibitory factor receptor (LIF-R)	Placebo	3.36 (0.27)	3.39 (0.23)	0.459	0.02 (0.19)	0.012
	Active	3.49 (0.30)	3.40 (0.20)	0.008	−0.10 (0.19)	
Macrophage colony-stimulating factor 1 (CSF-1)	Placebo	9.93 (0.39)	9.98 (0.37)	0.159	0.05 (0.21)	0.103
	Active	10.04 (0.27)	10.00 (0.20)	0.386	−0.03 (0.16)	
Matrix metalloproteinase-1 (MMP-1)	Placebo	15.26 (0.7)	15.23 (0.73)	0.380	−0.03 (0.21)	0.582
	Active	15.26 (0.82)	15.30 (0.80)	0.896	0.00 (0.19)	
Matrix metalloproteinase-10 (MMP-10)	Placebo	9.02 (0.48)	9.04 (0.44)	0.830	0.02 (0.44)	0.885
	Active	9.08 (0.48)	9.10 (0.70)	0.713	0.03 (0.50)	
Monocyte chemotactic protein 1 (MCP-1)	Placebo	13.18 (0.44)	13.30 (0.43)	0.148	0.12 (0.47)	0.381
	Active	13.10 (0.4)	13.10 (0.40)	0.568	0.03 (0.30)	
Monocyte chemotactic protein 2 (MCP-2)	Placebo	10.56 (0.63)	10.74 (0.83)	0.198	0.18 (0.79)	0.372
	Active	10.60 (0.84)	10.60 (0.80)	0.563	0.04 (0.36)	
Monocyte chemotactic protein 3 (MCP-3)	Placebo	1.70 (0.61)	1.84 (0.53)	0.148	0.14 (0.52)	0.239
	Active	1.76 (0.38)	1.70 (0.40)	0.957	0.00 (0.35)	
Monocyte chemotactic protein 4 (MCP-4)	Placebo	14.73 (0.70)	14.70 (0.68)	0.565	−0.03 (0.33)	0.445
	Active	14.90 (0.46)	14.80 (0.50)	0.093	−0.10 (0.30)	
Natural killer cell receptor 2B4 (CD244)	Placebo	5.89 (0.61)	5.92 (0.60)	0.361	0.03 (0.20)	0.124
	Active	5.94 (0.68)	5.90 (0.70)	0.228	−0.04 (0.16)	
Neurotrophin-3 (NT-3)	Placebo	2.43 (0.30)	2.48 (0.33)	0.330	0.05 (0.27)	0.238
	Active	2.63 (0.44)	2.60 (0.40)	0.490	−0.03 (0.26)	
Oncostatin-M (OSM)	Placebo	5.21 (0.74)	5.46 (0.81)	0.027	0.25 (0.63)	0.677
	Active	5.27 (0.98)	5.40 (0.80)	0.187	0.18 (0.73)	

Osteoprotegerin (OPG)	Placebo	10.49 (0.40)	10.58 (0.41)	0.134	0.09 (0.34)	0.052
	Active	10.60 (0.30)	10.60 (0.20)	0.188	−0.04 (0.17)	
Programmed cell death 1 ligand 1 (PD-L1)	Placebo	4.85 (0.33)	5.01 (0.38)	0.016	0.16 (0.36)	0.115
	Active	4.96 (0.27)	5.00 (0.30)	0.391	0.04 (0.23)	
Protein S100-A12 (EN-RAGE)	Placebo	4.77 (0.68)	5.24 (0.68)	0.002	0.47 (0.80)	0.353
	Active	4.88 (0.77)	5.10 (0.70)	0.061	0.28 (0.78)	
Signaling lymphocytic activation molecule (SLAMF1)	Placebo	1.99 (0.41)	2.04 (0.34)	0.305	0.05 (0.26)	0.308
	Active	2.04 (0.35)	2.00 (0.40)	0.694	−0.02 (0.23)	
SIR2-like protein 2 (SIRT2)	Placebo	2.39 (0.50)	2.52 (0.60)	0.059	0.17 (0.48)	0.460
	Active	2.33 (0.44)	2.60 (0.50)	0.013	0.27 (0.55)	
STAM-binding protein (STAMBP)	Placebo	3.64 (0.43)	3.75 (0.39)	0.111	0.11 (0.38)	0.973
	Active	3.71 (0.32)	3.80 (0.40)	0.178	0.11 (0.44)	
Stem cell factor (SCF)	Placebo	9.90 (0.28)	9.94 (0.24)	0.105	0.04 (0.13)	0.257
	Active	9.87 (0.32)	9.90 (0.30)	0.938	0.00 (0.15)	
Sulfotransferase 1A1 (ST1A1)	Placebo	2.19 (1.16)	1.75 (0.83)	0.111	−0.36 (1.10)	0.020
	Active	1.82 (0.82)	2.10 (1.00)	0.079	0.33 (0.86)	
T-cell surface glycoprotein CD5 (CD5)	Placebo	5.92 (0.40)	5.91 (0.37)	0.864	−0.01 (0.19)	0.092
	Active	6.02 (0.39)	5.90 (0.30)	0.017	−0.09 (0.20)	
T-cell surface glycoprotein CD6 isoform (CD6)	Placebo	5.32 (0.54)	5.36 (0.50)	0.272	0.04 (0.23)	0.110
	Active	5.43 (0.47)	5.40 (0.40)	0.249	−0.05 (0.23)	
T-cell surface glycoprotein CD8 alpha chain (CD8A)	Placebo	9.44 (0.53)	9.51 (0.60)	0.209	0.07 (0.32)	0.149
	Active	9.52 (0.42)	9.50 (0.40)	0.437	−0.04 (0.31)	

TNF-beta (TNFB)	Placebo	4.68 (0.50)	4.68 (0.53)	0.984	0.00 (0.33)	0.124
	Active	4.77 (0.30)	4.70 (0.30)	0.013	−0.11 (0.23)	
TNF-related activation-induced cytokine (TRANCE)	Placebo	4.15 (0.55)	3.99 (0.55)	0.019	−0.16 (0.37)	0.168
	Active	4.11 (0.69)	4.10 (0.60)	0.634	−0.03 (0.36)	
TNF-related apoptosis-inducing ligand (TRAIL)	Placebo	7.56 (0.34)	7.68 (0.43)	0.073	0.11 (0.35)	0.350
	Active	7.63 (0.29)	7.70 (0.30)	0.313	0.04 (0.23)	
Transforming growth factor alpha (TGF-α)	Placebo	3.34 (0.65)	3.57 (0.80)	0.032	0.22 (0.57)	0.518
	Active	3.47 (0.78)	3.50 (0.60)	0.164	0.13 (0.50)	
Tumor necrosis factor (ligand) superfamily, member 12 (TWEAK)	Placebo	9.10 (0.34)	9.08 (0.33)	0.658	−0.01 (0.18)	0.033
	Active	9.22 (0.26)	9.10 (0.20)	0.002	−0.12 (0.19)	
Tumor necrosis factor (TNF)	Placebo	3.71 (0.56)	3.86 (0.67)	0.085	0.15 (0.50)	0.142
	Active	3.76 (0.41)	3.80 (0.40)	0.893	0.01 (0.23)	
Tumor necrosis factor ligand superfamily member 14 (TNFSF14)	Placebo	6.08 (0.89)	6.11 (0.76)	0.845	0.02 (0.73)	0.521
	Active	6.04 (0.76)	6.20 (0.80)	0.321	0.15 (0.82)	
Tumor necrosis factor receptor superfamily member 9 (TNFRSF9)	Placebo	5.73 (0.54)	5.75 (0.51)	0.679	0.02 (0.28)	0.488
	Active	5.79 (0.41)	5.80 (0.40)	0.542	−0.02 (0.21)	
Urokinase-type plasminogen activator (uPA)	Placebo	10.34 (0.28)	10.41 (0.37)	0.167	0.06 (0.26)	0.212
	Active	10.51 (0.34)	10.50 (0.30)	0.781	−0.01 (0.20)	
Vascular endothelial growth factor A (VEGF-A)	Placebo	11.76 (0.62)	11.82 (0.63)	0.134	0.06 (0.24)	0.483
	Active	12.09 (0.60)	12.10 (0.60)	0.641	0.02 (0.24)	

All data were obtained using the Olink® Target 96 Inflammation Panels and are presented as the mean (SD) of log base 2 normalized protein expression values.
Data at week 12 were compared with those at week 0, using the paired *t*-test.
Comparisons between the placebo and active groups were tested by the unpaired *t*-test.
