# Supplementary material for: Effect of Continuous Ingestion of Bifidobacteria and Dietary Fiber on Improvement in Cognitive Function: A Randomized, Double-Blind, Placebo-Controlled Trial
Source: Nutrients. 2023 Sep 27;15(19):4175. doi: 10.3390/nu15194175 (PMC10574581; doi:10.3390/nu15194175)
Supplement: Supplementary file 1 [file nutrients-15-04175-s001.zip › Supplementary materials/Supplementary Table S4.pdf]

Table S4. Post-intervention changes in IL-1β and *hs*-CRP scores.

		Week 0	Week 12		Change	
		Mean (SD)	Mean (SD)	*P*−value	Mean (SD)	*P*−value
IL-1β, fg/mL	Placebo	69.25 (57.37)	69.3 (43.9)	0.283	9.34 (46.01)	0.584
	Active	65.86 (40.05)	94.8 (185.2)	0.407	28.73 (183.68)	
hs-CRP, mg/dL	Placebo	0.04 (0.04)	0.06 (0.07)	0.071	0.02 (0.06)	0.577
	Active	0.08 (0.16)	0.14 (0.36)	0.394	0.06 (0.37)	

Data at week 12 were compared with those at week 0, using the paired *t*-test.

Comparisons between the placebo and active groups were tested by the unpaired *t*-test.
